# Development and evaluation of antibody-capture immunoassays for detection of Lassa virus nucleoprotein-specific immunoglobulin M and G

**DOI:** 10.1371/journal.pntd.0006361

**Published:** 2018-03-29

**Authors:** Martin Gabriel, Donatus I. Adomeh, Jacqueline Ehimuan, Jennifer Oyakhilome, Emmanuel O. Omomoh, Yemisi Ighodalo, Thomas Olokor, Kofi Bonney, Meike Pahlmann, Petra Emmerich, Michaela Lelke, Linda Brunotte, Stephan Ölschläger, Corinna Thomé-Bolduan, Beate Becker-Ziaja, Carola Busch, Ikponmwosa Odia, Ephraim Ogbaini-Emovon, Peter O. Okokhere, Sylvanus A. Okogbenin, George O. Akpede, Herbert Schmitz, Danny A. Asogun, Stephan Günther

**Affiliations:** 1 Department of Virology, Bernhard Nocht Institute for Tropical Medicine, Hamburg, Germany; 2 Institute of Lassa Fever Research and Control, Irrua Specialist Teaching Hospital, Irrua, Edo State, Nigeria; 3 Department of Virology, Noguchi Memorial Institute for Medical Research, University of Ghana, Legon, Ghana; 4 Department of Tropical Medicine and Infectious Diseases, Center of Internal Medicine II, University of Rostock, Rostock, Germany; 5 Institute of Molecular Virology, Westfälische Wilhelms University of Münster, Münster, Germany; 6 Department of Medicine, Irrua Specialist Teaching Hospital, Irrua, Edo State, Nigeria; 7 Department of Obstetrics and Gynecology, Irrua Specialist Teaching Hospital, Irrua, Edo State, Nigeria; 8 Department of Pediatrics, Irrua Specialist Teaching Hospital, Irrua, Edo State, Nigeria; 9 German Center for Infection Research (DZIF), Partner Site Hamburg–Lübeck–Borstel–Riems, Germany; Aix Marseille University, Institute of Research for Development, and EHESP School of Public Health, FRANCE

## Abstract

**Background:**

The classical method for detection of Lassa virus-specific antibodies is the immunofluorescence assay (IFA) using virus-infected cells as antigen. However, IFA requires laboratories of biosafety level 4 for assay production and an experienced investigator to interpret the fluorescence signals. Therefore, we aimed to establish and evaluate enzyme-linked immunosorbent assays (ELISA) using recombinant Lassa virus nucleoprotein (NP) as antigen.

**Methodology/Principal findings:**

The IgM ELISA is based on capturing IgM antibodies using anti-IgM, and the IgG ELISA is based on capturing IgG antibody–antigen complexes using rheumatoid factor or Fc gamma receptor CD32a. Analytical and clinical evaluation was performed with 880 sera from Lassa fever endemic (Nigeria) and non-endemic (Ghana and Germany) areas. Using the IFA as reference method, we observed 91.5–94.3% analytical accuracy of the ELISAs in detecting Lassa virus-specific antibodies. Evaluation of the ELISAs for diagnosis of Lassa fever on admission to hospital in an endemic area revealed a clinical sensitivity for the stand-alone IgM ELISA of 31% (95% CI 25–37) and for combined IgM/IgG detection of 26% (95% CI 21–32) compared to RT-PCR. The specificity of IgM and IgG ELISA was estimated at 96% (95% CI 93–98) and 100% (95% CI 99–100), respectively, in non-Lassa fever patients from non-endemic areas. In patients who seroconverted during follow-up, Lassa virus-specific IgM and IgG developed simultaneously rather than sequentially. Consistent with this finding, isolated IgM reactivity, i.e. IgM in the absence of IgG, had no diagnostic value.

**Conclusions/Significance:**

The ELISAs are not equivalent to RT-PCR for early diagnosis of Lassa fever; however, they are of value in diagnosing patients at later stage. The IgG ELISA may be useful for epidemiological studies and clinical trials due its high specificity, and the higher throughput rate and easier operation compared to IFA.

## Author summary

Lassa fever is endemic in several West African countries. However, only few hospitals and laboratories in the region have the capacity to conduct molecular or serological Lassa fever diagnostics. One reason is that the classical serological technique for Lassa fever—the immunofluorescence assay (IFA)—requires biosafety level 4 laboratories, which are not available in the Lassa fever endemic countries. In addition, IFA does not feature an objective read-out. Therefore, we established enzyme-linked immunosorbent assays (ELISA) for detection of Lassa virus-specific IgM and IgG in 96-well format, which do not require expensive equipment and can be implemented in diagnostic laboratories in West Africa. The ELISAs are based on recombinant antigen facilitating future production according to industry standards. In our evaluation, the ELISAs have shown a performance comparable to IFA. They allow in particular the diagnosis of Lassa fever patients at later stages of the acute illness. In addition, reliable serological assays, such as those described here, are urgently needed to conduct large-scale epidemiological investigations to better understand the epidemiology of Lassa fever across West Africa as well as for clinical trials evaluating novel medical countermeasures including vaccines and drugs.

## Introduction

Lassa fever is a viral hemorrhagic fever endemic in West Africa [1–14]. The causative agent is Lassa virus, an RNA virus of the family *Arenaviridae*. Its main natural host is the rodent *Mastomys natalensis* [15, 16]. Contamination of households and food by rodent excreta is a likely mode of virus transmission to humans. The virus may be further transmitted from human to human and cause epidemics mainly in the nosocomial setting [2, 3, 5, 17]. However, the Lassa virus-specific seroprevalence in endemic areas is high, indicating that most Lassa virus infections in the communities are probably mild [4].

Clinically, Lassa fever mainly presents with flu-like and gastrointestinal symptoms and is difficult to distinguish from other febrile illnesses seen in West African hospitals [7, 18, 19]. Therefore, diagnosis requires laboratory confirmation. Reverse transcription polymerase chain reaction (RT-PCR) and antigen detection are valuable tools for early diagnosis of Lassa fever [12, 20–26]. IgM and IgG antibodies are detectable in a fraction of patients during the first days of illness. While patients with fatal Lassa fever do not necessarily develop antibodies [27, 28], they are commonly detectable in survivors [20–22, 29]. The classical method for detection of Lassa virus-specific antibodies is immunofluorescence assay (IFA) using virus-infected cells as antigen [27, 30]. However, this method requires laboratories of biosafety level 4 for virus propagation and an experienced investigator to interpret the fluorescence signals.

The aim of this study was the establishment and evaluation of IgM and IgG enzyme-linked immunosorbent assays (ELISA) based on recombinant Lassa virus nucleoprotein (NP). We have chosen NP, as previous work provided evidence that ELISA methods detecting the NP-specific antibody response compare favorably with the conventional methods for the serological diagnosis of Lassa fever [31, 32]. Our IgM assay is based on capturing IgM antibodies with human anti-μ chain antibodies on the solid phase (IgM μ-capture ELISA). The test principle of our IgG assay is based on capturing IgG antibody–antigen complexes via rheumatoid factor (RF), a human anti-IgG autoantibody, or via human Fc gamma receptor molecule CD32a on the solid phase (IgG RF ELISA and IgG CD32 ELISA). Both RF and CD32 preferentially bind antibodies in complex with antigen, which facilitates sensitive detection of specific IgG in the capture assay [33–36].

## Methods

### Ethics statement

Ethical permission was granted by the Research and Ethics Committee of Irrua Specialist Teaching Hospital (ISTH), Edo State, Nigeria (ISTHREC09/14/12/2009), the Ärztekammer Hamburg, Germany (PV3187), and the Institutional Review Board of the Noguchi Memorial Institute for Medical Research, Ghana (NMIMR-IRB 003/07-08). Written informed consent was obtained from all study subjects. If study participants were minors, the parents/guardians provided consent on behalf of all child participants. In addition, leftover specimens, i.e. remnants of specimens collected for routine clinical care that would otherwise have been discarded, were included in the assay evaluation. These specimens were anonymized and used without informed consent.

### Patients and specimens

A total of 576 sera from the diagnostic service of the Institute of Lassa Fever Research and Control at ISTH, which is located in a Lassa fever endemic zone in Edo State, Nigeria, were chosen for the study; 394 sera were left over from the first specimen of patients with suspected Lassa fever that was sent to the laboratory for Lassa virus diagnostics between 2008 and 2011 (also called diagnostic specimen) [10]. Of those, 270 sera tested positive by Lassa virus RT-PCR [25] establishing the diagnosis of Lassa fever; 101 sera tested negative by Lassa virus RT-PCR; and 23 had no RT-PCR result. From 47 RT-PCR confirmed Lassa fever patients, 182 (1–9 per patient) follow-up sera were available. Three follow-up specimens were excluded from further analysis, as the data obtained with these specimens were implausible within the context of the data obtained with other follow-up specimens of the respective patients (S1 Data), suggesting mix-up of samples during sampling or processing. The diagnostic specimen was missing for one follow-up patient.

From Lassa fever non-endemic areas, 199 samples collected between 2008 and 2011 from patients with suspected viral hemorrhagic fever or viral hepatitis in Ghana, all of whom tested negative by Lassa virus RT-PCR [37], and 105 diagnostic leftover samples from German patients with various unknown diseases were included in the study. The travel history of the patients from non-endemic areas was not known.

The specimens for this study were randomly chosen from the available specimen pools. However, to provide a more meaningful collection for the purpose of this study, patients who tested positive in RT-PCR were intentionally overrepresented. Thus, the prevalence of Lassa fever patients among the study population does not reflect the true prevalence of Lassa fever patients among all patients tested at ISTH. All samples were analyzed retrospectively and therefore, the information generated in this study was not known to those who performed the RT-PCR assays. The investigator who performed IFA and ELISA knew the origin of the samples during evaluation, though not the RT-PCR result. IFA and ELISA were performed in a blinded fashion, i.e. the investigator did not know the corresponding assay result. Samples from each setting (Nigeria, Ghana, Germany) were tested consecutively according to the identification number. Diagnosis (Lassa fever or non-Lassa fever), IFA result, and ELISA result were linked after testing.

Presence and absence of disease were defined as follows: Presence of Lassa fever was defined by a positive result in the Lassa virus RT-PCR on the diagnostic specimen. RT-PCR was chosen as reference standard, as it is the method of choice for early diagnosis of Lassa fever [20, 21]. Details on the RT-PCR assay used in our study have been published previously [10, 25]. Absence of Lassa fever was primarily defined by origin of the patient from a non-endemic area. As sporadic Lassa fever cases in Ghana cannot definitively be excluded, classification as "non-Lassa fever" in Ghana included a negative RT-PCR result. Due to the definitive absence of Lassa fever and the virus reservoir in Germany, patients from this country were defined as "non-Lassa fever" without further laboratory testing. RT-PCR-negative patients from Nigeria were not classified as "non-Lassa fever", as a negative RT-PCR result does not rule out Lassa fever in the endemic area, for example if the patients present at late stage or have a mild course of disease. Because of this ambiguity and because the high prevalence of pre-existing Lassa virus IgG antibodies in these patients would hamper estimation of assay specificity, they were not included in the analysis of clinical accuracy. In summary, origin of patient from a non-endemic country in combination with negative RT-PCR was chosen as reference to define absence of disease, as this deemed more accurate in ruling out Lassa virus infection than a negative RT-PCR result in a patient from endemic area. All Nigerian samples were used to estimate analytical performance using IFA as reference method. IFA was chosen as analytical reference, as it is the classical test for detection of Lassa virus-specific antibodies in patient sera and the diagnostic standard test in our laboratory [27, 30].

We aimed at a sample size of approximately 300 patients per group, as this number facilitates detection of small proportions at reasonable precision, for example a 2%-false positive rate with specified limits of the 95% confidence interval at 0.5% and 3.5% (http://sampsize.sourceforge.net/iface/index.html#prev). Information on compliance of this study with standards for reporting diagnostic accuracy studies is given in S1 Checklist and S1 Diagram. The data generated in this study are listed in S1 Data.

### Lassa virus-specific IFA

Lassa virus strain AV [38] was propagated in Vero cells in the biosafety level 4 facility, Hamburg, Germany. Infected cells were spread onto immunofluorescence slides, air-dried, and acetone-fixed. Serum samples were diluted 1:20 and 1:80 and incubated for 2 h with the cells. After washing with phosphate buffered saline (PBS), bound antibodies were detected by anti-human IgG or IgM labeled with fluorescein isothiocyanate (Dianova). Signals were evaluated by fluorescence microscopy and classified as "clearly negative", "probable positive", and "clearly positive" by the investigator. For consistency, a single investigator evaluated all slides.

### Expression and purification of recombinant NP

*Spodoptera frugiperda* (Sf9) cells (Invitrogen) in several T75 cell culture flasks were inoculated with a high-titered stock of recombinant baculovirus expressing NP of Lassa virus strain AV (GenBank no. AAG41803) fused to C-terminal FLAG-6xHis sequence [38, 39]. Six days post infection, cells were lysed in 50 mM NaH_2_PO_4_ (pH 8)–500 mM NaCl–10 mM imidazole–0.5% NP-40–1x Complete Protease Inhibitor Cocktail (Roche)–25 U/ml Benzonase (Novagen) and treated by sonication. Cell debris was removed by centrifugation and the supernatant was incubated with Ni-NTA agarose (Invitrogen) overnight at 4°C. The agarose was washed 5 times with 50 mM NaH_2_PO_4_ (pH 8)–500 mM NaCl–30 mM imidazole and NP was eluted in 50 mM NaH_2_PO_4_ (pH 8)–500 mM NaCl–500 mM imidazole. Purity of the protein was verified by polyacrylamide gel electrophoresis. NP was dialyzed overnight against PBS, quantified by Bradford assay, and about 0.4 mg were biotinylated using EZ-Link Sulfo-NHS-Biotin reagent (Thermo Fisher) at 20-fold molar excess on ice for 2 h according to the manufacturer’s instructions. The protein was desalted using Zeba Spin Desalting Columns (7K MWCO, 2 ml; Thermo Fisher) and stored 1:1 in glycerol at –20°C. For use as antigen in ELISA, biotinylated NP (100–300 μg/ml) was diluted 1:200–1:800 in 1% Triton X-100–1% bovine serum albumin–PBS. Optimal dilutions for each lot of antigen were determined empirically.

### Lassa virus NP-specific IgG and IgM ELISA

ELISA plates (Nunc Thermo Scientific 469949, Immuno Clear Standard Modules, F8, MaxiSorp) were coated with recombinant human Fc gamma RIIA/CD32a (R&D Systems 1330-CD-050/CF) at a concentration of 8 μg/ml in PBS–0.01% sodium azide for 3–5 days at 4°C and washed three times with 0.05% Tween 20–PBS before use (CD32-coated IgG ELISA plates). ELISA plates coated with rheumatoid factor (4 μg/well), an autoantibody prepared from blood of patients with rheumatoid arthritis that is directed against the Fc portion of IgG, were custom-made by Medac Company, Hamburg, Germany (RF-coated IgG ELISA plates). ELISA plates coated with anti-human IgM antibodies were also obtained from Medac (μ-capture IgM ELISA plates).

For detection of NP-specific IgG, serum diluted 1:20 in 1% Triton X-100–PBS was mixed 1:1 with biotinylated NP antigen, and 50 μl thereof were incubated in a well of a CD32- or RF-coated ELISA plate overnight at 4°C. For detection of NP-specific IgM, 50 μl serum diluted 1:20 in 1% Triton X-100–PBS was incubated in a well of a μ-capture ELISA plate for 2 h at 4°C; then the wells were washed three times with 0.05% Tween 20–PBS and incubated with 50 μl biotinylated NP antigen overnight at 4°C. Following incubation with serum and antigen, ELISA plates were washed three times with 0.05% Tween 20–PBS and incubated with 50 μl streptavidin–horseradish peroxidase conjugate (1:1,000–1:2,000 in PBS; Sigma-Aldrich) per well for 1 h at 4°C. Wells were washed three times with 0.05% Tween 20–PBS and incubated with 50 μl 3,3',5,5'-tetramethylbenzidine substrate for 10 minutes. The reaction was stopped by adding 50 μl 0.5 M H_2_SO_4_ and the optical density (OD) was measured in a 96-well plate reader at 450/630 nm.

A variety of arbitrary formulas exist for calculation of ELISA cut-off, most of which are based on negative controls [40]. Our cut-off determination considered the following aspects: (i) The cut-off is calculated using negative controls, which can be easier generated and replenished compared to positive control sera with a specific titer. (ii) The formula does not include the standard deviation of negative controls, as reliable estimation of this parameter requires testing of a larger number of negative controls on each ELISA plate. (iii) To account for differences in the end-point of the colorimetric reaction on individual ELISA plates, the cut-off is proportional to the mean OD of negative controls (scale factor *a*). (iv) A constant (*b*) serves as baseline cut-off. According to these criteria, we used the formula: *Cut-off = a × Mean OD of negative standards + b*, which is a modification of the formulas F_1_ and F_2_ in Lardeux et al. [40]. Three representative negative sera were selected as standards for cut-off determination and included in all assays performed in this study. After all data had been collected, we found empirically that *a = 3* and *b = 0*.*06* facilitate a good correlation between ELISA and IFA results independent of the colorimetric end-point of a plate, in particular for sera classified as "clearly negative" or "clearly positive" in IFA. Except for outliers in the IgM ELISA, sera from non-endemic countries were negative according to this formula. Cut-off values ranged from 0.094 to 0.457 reflecting the plate-specific colorimetric end-points. The cut-off and sample OD values for individual ELISA plates are shown in S1 Fig. Each assay also included two positive control sera. The reactivity of a serum sample was expressed as sample to cut-off ratio (S/CO) or log_10_ S/CO. Samples with S/C > 1 or log_10_ S/CO > 0, respectively, were considered positive.

### Calculation of performance characteristics

Analytical performance characteristics of the ELISAs were calculated as follows:

Sensitivity=NpositivebothbyIFAandELISA/NpositivebyIFA

Specificity=NnegativebothbyIFAandELISA/NnegativebyIFA

PositivePredictiveValue(PPV(=NpositivebothbyIFAandELISA/NpositivebyELISA

NegativePredictiveValue(NPV(=NnegativebothbyIFAandELISA/NnegativebyELISA

Accuracy=(NpositivebothbyIFAandELISA+NnegativebothbyIFAandELISA(/Nalltested

The term *positive* may also represent a specific serological constellation arising from the combined use of IgM and IgG assays, i.e. IgM+/IgG+, IgM+/IgG–, or IgM–/IgG+.

Clinical performance characteristics of the ELISA for diagnosis of acute Lassa fever, such as sensitivity, specificity, positive likelihood ratio, and negative likelihood ratio including 95% confidence intervals (95% CI) were estimated using the statistical calculator at https://www.medcalc.org/calc/diagnostic_test.php. Calculation of clinical PPV and NPV considered the prevalence of Lassa fever patients among all patients with suspected Lassa fever tested (*LF Prevalence*):

PPV=Sensitivity×LFPrevalence/[Sensitivity×LFPrevalence+(1–Specificity(×(1–LFPrevalence)]

NPV=Specificity×(1−LFPrevalence(/[(1–Sensitivity)×LFPrevalence+Specificity×(1–LFPrevalence)]

Sensitivity=NLassafeverpatientspositivebyELISA/NallLassafeverpatients

Specificity=Nnon-LassafeverpatientsnegativebyELISA/Nallnon-Lassafeverpatients

The specificity for combined use of IgM and IgG ELISA was calculated as: *Specificity of combined test = 1 –False Positive Rate of IgM ELISA × False Positive Rate of IgG ELISA*. Pre-existing Lassa virus-specific IgG among patients with suspected Lassa fever (*IgG Prevalence)* was considered in the false positive rate of the IgG ELISA, as IgG from past infection occurs exclusively in non-Lassa fever patients (there is no evidence for secondary Lassa fever): *False Positive Rate of IgG ELISA = IgG Prevalence / (1 –LF Prevalence)*. To simplify calculation, we assumed a linear 1:3 relationship between prevalence of Lassa fever patients and IgG seroprevalence: *IgG Prevalence = 3 × LF Prevalence*.

## Results

**Fig 1:**
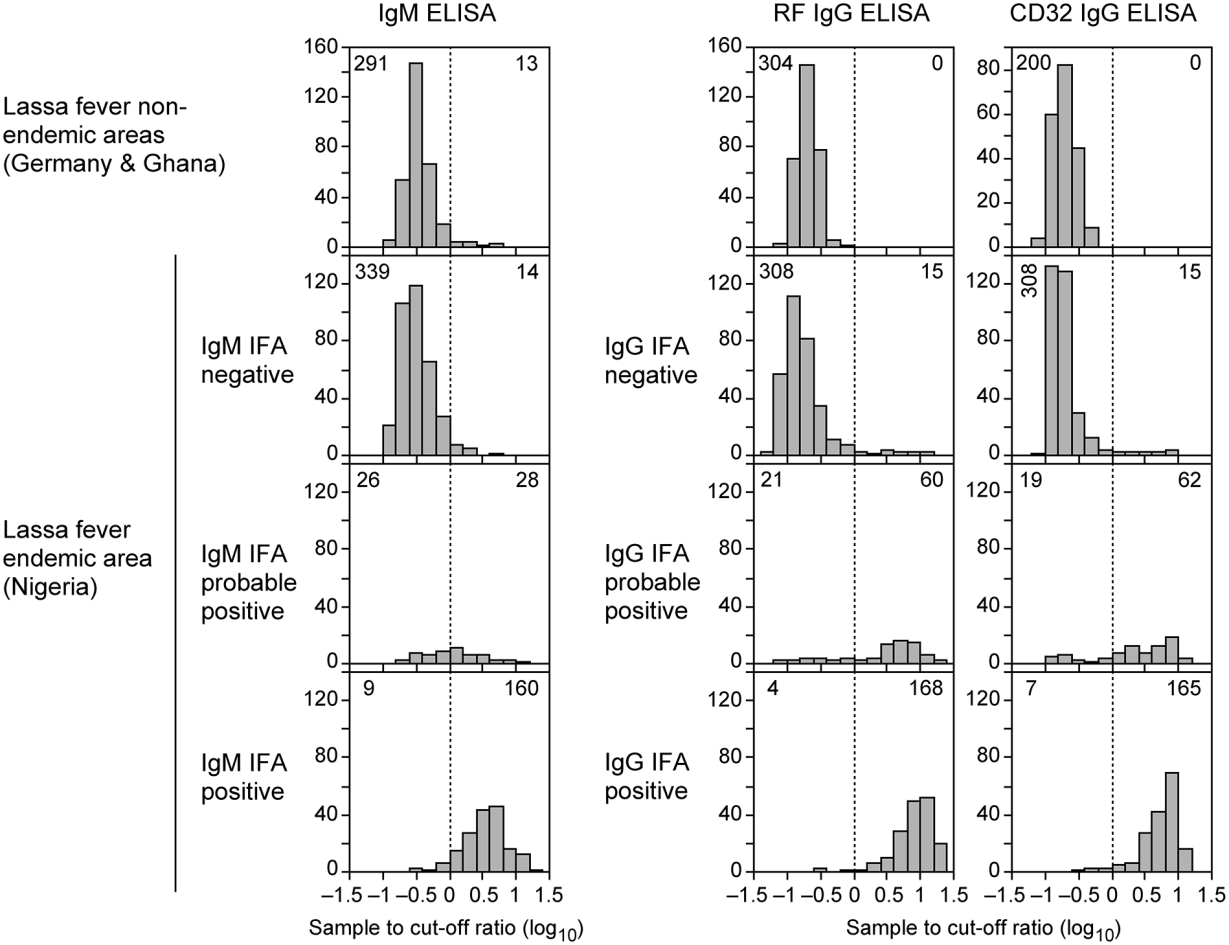
Sample-to-cut-off (S/CO) values for 880 sera tested in IgM ELISA, RF-based IgG ELISA, and CD32-based IgG ELISA and comparison with IFA results. The S/CO values obtained with the ELISA are shown as histograms according to sample origin, type of ELISA, and IFA results. The cut-offs for the ELISAs are indicated by vertical dotted lines. The number of samples with OD < CO and OD > CO is indicated in left and right corner, respectively, of each diagram.

**Fig 2:**
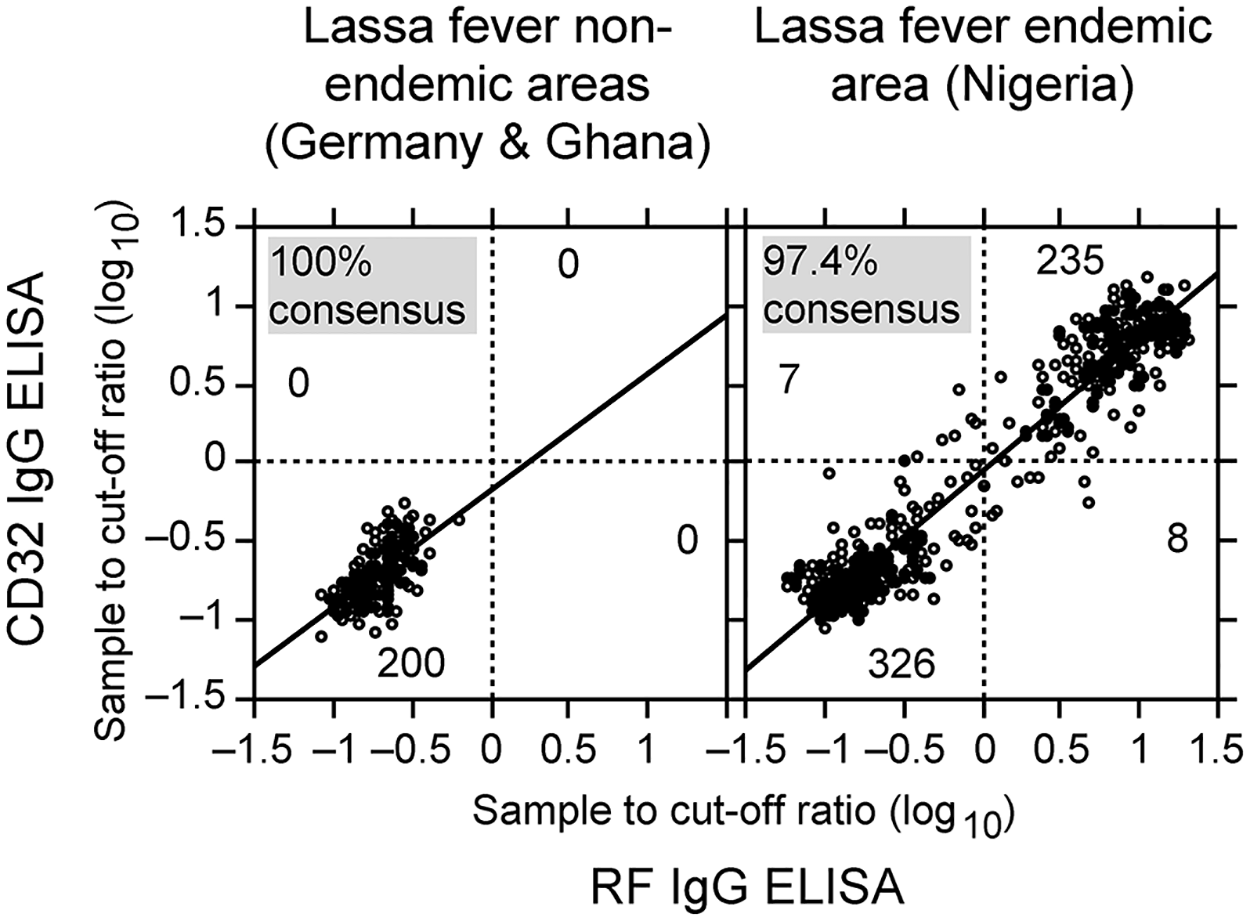
Comparison of RF- and CD32-based IgG ELISA results obtained from 776 sera. The S/CO values obtained with the RF ELISA were plotted against the S/CO values of the CD32 ELISA. The cut-offs for both assays are indicated by horizontal and vertical dotted lines. The number of samples per quadrant is also given. The regression curve is shown as straight line. Samples from Lassa fever non-endemic and endemic countries were plotted separately.

Both IgG and IgM ELISA were designed as indirect capture assays as described previously [32–36]. The IgM ELISA plates were coated with anti-human IgM for capturing IgM antibodies and the IgG ELISA plates were coated with RF or human Fc gamma receptor CD32a for capturing specific IgG antibody–antigen complexes. Lassa virus NP was expressed in insect cells and used as antigen. NP was labeled with biotin for detection of antibody–antigen complexes via streptavidin–peroxidase conjugate. The concentrations of the ELISA components were adjusted so that negative control sera yielded OD values between 0.02 and 0.08 and positive control sera yielded OD values between 1.0 and 2.5. Cut-off values typically ranged from 0.15–0.3. All experiments were performed with the μ-capture IgM ELISA, the RF-based IgG ELISA, and the CD32-based IgG ELISA. The sample to cut-off (S/CO) values obtained for the 880 sera included in this study are summarized in Fig 1. A comparison of the S/CO values of the RF-based vs. the CD32-based IgG ELISA revealed a high degree of congruence between both read-outs (Fig 2), indicating that technically both assays are comparable.

The analytical performance of the ELISAs to detect Lassa virus-specific antibodies was first evaluated in comparison to IFA as serological reference method. The test panel included 576 sera from a hospital in a Lassa fever endemic area in Nigeria. All sera were tested by IgM and IgG IFA and classified as "clearly negative", "probable positive", and "clearly positive". The S/CO values obtained with IgM, RF IgG, and CD32 IgG ELISA correlated well with the IFA categories "clearly negative" and "clearly positive" resulting in ≥95% correspondence of ELISA and IFA results (Fig 1). As expected, the S/CO range of sera classified as "probable positive" in IFA was broader, though for the IgG ELISAs the S/CO ratios in this category tended to be >1 (Fig 1). For calculation of analytical performance characteristics of the ELISAs, the "probable" and "clearly positive" IFA categories were merged into one "positive" category. The analytical specificity of the ELISAs ranged from 95–98% for all assay combinations (Table 1). The analytical sensitivity of the individual IgM and IgG ELISA was 84% and 90%, respectively, indicating good agreement with IFA in detecting positive samples. When IgM and IgG assays were used in combination, the analytical sensitivity depended on the serological constellation (Table 1). There was poor agreement of the ELISAs with IFA in detecting the IgM+/IgG–status (analytical sensitivity 12.5%), while the IgM+/IgG+ status was detected with a sensitivity of 87%. In summary, there was good analytical performance of the ELISAs in detecting Lassa virus-specific IgM and IgG antibodies or antibody combinations, except for the IgM+/IgG–status.

**Table 1:** Analytical performance of IgM and IgG ELISA in detecting Lassa virus-specific antibodies in 576 serum samples from a Lassa fever endemic area in Nigeria using IFA as a reference method.

Serological status defined by IFA	Type of IgG ELISA	Performance of ELISA vs. IFA								
		True pos, n	False pos, n	True neg, n	False neg, n	Specificity, %	Sensitivity, %	PPV, %	NPV, %	Accuracy, %
IgM+	None	188	14	339	35	96.0	84.3	93.1	90.6	91.5
IgG+	RF	228	15	308	25	95.4	90.1	93.8	92.5	93.1
	CD32	227	15	308	26	95.4	89.7	93.8	92.2	92.9
IgM+ / IgG–	RF	3	15	537	21	97.3	12.5	16.7	96.2	93.8
	CD32	3	18	534	21	96.7	12.5	14.3	96.2	93.2
IgM–/ IgG+	RF	39	20	502	15	96.2	72.2	66.1	97.1	93.9
	CD32	41	20	502	13	96.2	75.9	67.2	97.5	94.3
IgM+ / IgG+	RF	174	10	367	25	97.3	87.4	94.6	93.6	93.9
	CD32	171	10	367	28	97.3	85.9	94.5	92.9	93.4

Abbreviations: ELISA enzyme-linked immunosorbent assay; IFA, immunofluorescence assay; NPV, negative predictive value; PPV, positive predictive value; RF, rheumatoid factor.

**Fig 3:**
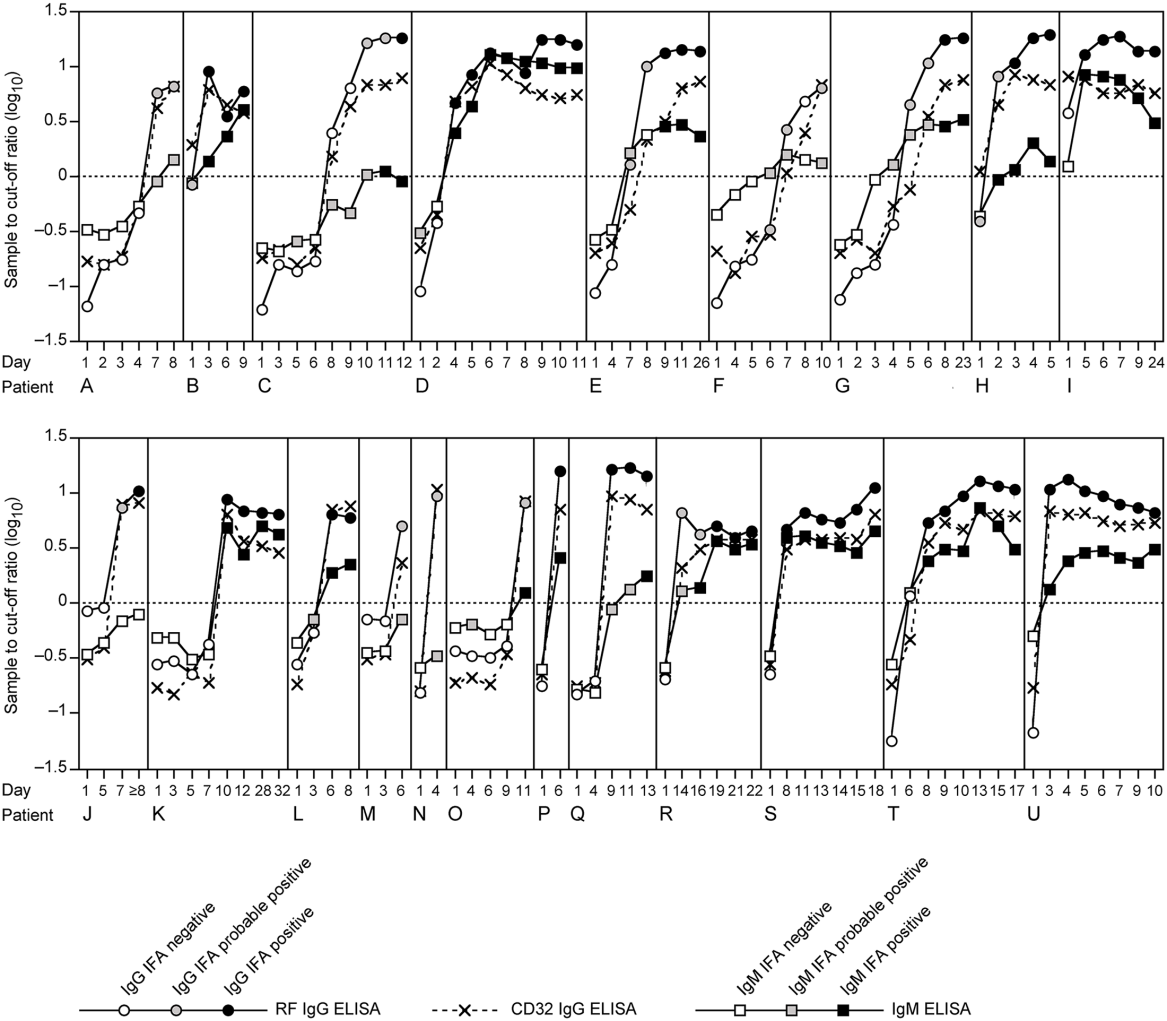
IgM and/or IgG seroconversion detected by ELISA and IFA during follow-up of 21 Lassa fever patients. Each of the panels A–U depicts an individual patient. The cut-off for the ELISAs is indicated by a horizontal dotted line. The timeline for each patient is given in days with day 1 being the day the first sample was taken for Lassa fever diagnostics. IFA results are coded by symbols according to the categories "clearly negative", probable positive", and "clearly positive" on the S/CO curves of the corresponding ELISA.

The clinical evaluation of the ELISAs was performed using sera from 270 Lassa fever patients. About one third of these patients was IgM-positive in the first—diagnostic—specimen, i.e. the specimens that had been used to establish the diagnosis by Lassa virus RT-PCR (Table 2). The majority (83%) of IgM positives was also positive for IgG and only a minority (17%) had isolated IgM. Follow-up sera were available for 47 Lassa fever patients. The percentage of IgM positive as well as IgM/IgG positive sera among these follow-up sera was about 60%, while isolated IgM was less prevalent than in the first specimen (Table 2). The low prevalence of patients with isolated IgM compared to patients showing IgM as well as IgG suggested that both antibody classes develop concurrently in the majority of Lassa fever patients. This conclusion was further substantiated in 21 patients, who seroconverted during follow-up. In 12/21 (57%) IgM and IgG emerged at the same time (Fig 3, patients B, D, E, I, K, L, O, P, R, S, T, U). In 7/12 (33%), IgM seroconversion was delayed or absent relative to IgG seroconversion (Fig 3, patients A, C, H, J, M, N, Q). Only in 2/21 (9.5%) (Fig 3, patients F and G), IgM emerged shortly before IgG. There was no significant difference between ELISA and IFA regarding the time point when seroconversion was detected. However, IgG IFA results around seroconversion often scored "probable positive", while the IgG ELISAs already showed clear positive results with S/CO ratios >>1 (Fig 3).

**Table 2:** IgM and IgG ELISA results in patients grom various settings.

Clinical condition and setting	Type of IgG ELISA	No. samples / patients tested	ELISA result (% samples)				
			IgM+^a^	IgM+ only^b^	IgM+ and IgG+^c^	IgG+^d^	IgG+ only^e^
Clinical Lassa fever suspicion and Lassa virus RT-PCR-positive on diagnostic specimen in endemic area (Nigeria)	RF	270/270	31.1	5.2	25.9	33.7	7.8
	CD32	270/270	31.1	5.2	25.9	33.3	7.4
Follow-up specimens of Lassa virus RT-PCR-positive patients (Nigeria)	RF	179/47	62.0	1.7	60.3	71.5	11.2
	CD32	179/47	62.0	3.4	58.7	69.8	11.2
Clinical Lassa fever suspicion and Lassa virus RT-PCR-negative in endemic area (Nigeria)	RF	101/101	5.0^f^	1.0	4.0^f^	20.8	16.8
	CD32	101/101	5.0^f^	1.0	4.0^f^	23.8	19.8
Clinical VHF suspicion and Lassa virus RT-PCR-negative in non-endemic area (Ghana)	RF	199/199	6.5^g^	6.5	0	0	0
	CD32	100/100	7.0^g^	7.0	0	0	0
Patients with various diseases in non-endemic area (Germany)	RF	105/105	0	0	0	0	0
	CD32	100/100	0	0	0	0	0

Abbreviations: ELISA enzyme-linked immunosorbent assay; RF, rheumatoid factor; VHF, viral hemorrhagic fever.

^a^ Sample was IgM positive irrespective of IgG result.

^b^ Sample was IgM positive and IgG negative.

^c^ Sample was IgM positive and IgG positive.

^d^ Sample was IgG positive irrespective of IgM result.

^e^ Sample was IgG positive and IgM negative.

^f^ All IgM and IgG positive samples also tested positive for IgM and IgG, respectively, in IFA.

^g^ All IgM positive samples tested negative for IgM in IFA.

Nearly 20% of Lassa virus RT-PCR-negative patients from the endemic area in Nigeria—pre-classified as inconclusive Lassa fever status—showed isolated IgG reflecting the seroprevalence in the area (Table 2). Five percent were found positive in IgM ELISA and most of those were also positive in IgG ELISA (Table 2). These findings were confirmed by IFA, suggesting that the IgM-positive patients might have been in convalescence phase or had Lassa fever recently.

Sera from non-Lassa fever patients provided information on the specificity of the ELISA. All non-Lassa fever patients from Ghana (Lassa virus RT-PCR negative) and Germany were negative in IgG ELISA (Table 2). The patients from Germany were also negative in IgM ELISA, while 13 sera from Ghana showed a positive signal in IgM ELISA (Table 2). However, the latter sera tested negative for IgM in IFA and showed a positive signal in ELISA even in absence of NP antigen, indicating that the signals result from unspecific binding. Therefore, these samples were classified as IgM false positives.

Based on the data presented in Table 2, we estimated the performance characteristics of the ELISAs for diagnosis of acute Lassa fever in the first specimen taken from a patient (Table 3). The characteristics were calculated for the IgM ELISA as stand-alone test and in combination with the IgG ELISA. Due to the high seroprevalence in endemic areas as described here and elsewhere [4, 7], the IgG ELISA was considered for diagnostics of acute patients only in combination with the IgM ELISA. The sensitivity of the IgM ELISA was 31% irrespective of the IgG result; 5.2% for isolated IgM detection (IgM+/IgG–); and 26% for detection of both IgM and IgG (IgM+/IgG+). The specificity of the IgM ELISA was estimated to be 96% based on the frequency of false positive reactions among non-Lassa fever patients from non-endemic areas. The specificity of the IgG ELISAs was estimated to be 100%, as there was no evidence for false positive reactions among patients from non-endemic areas. The likelihood ratios indicate good diagnostic value for the stand-alone IgM ELISA and combined IgM and IgG detection (IgM+/IgG+). However, isolated IgM detection (IgM+/IgG–) has no diagnostic value, as the likelihood ratios are close to 1 and their 95% CI overlap with 1 (Table 3).

**Table 3:** Clinical performance characteristics of the IgM ELISA as stand-alone test and in combination with the IgG ELISA for early diagnosis of Lassa fever.

	IgM^a^	RF IgG ELISA		CD32 IgG ELISA	
		IgM only^b^	IgM and IgG^c^	IgM only^b^	IgM and IgG^c^
Lassa fever patients^d^, n	270	270	270	270	270
Test positive, n	84	14	70	14	70
Test negative, n	186	256	200	256	200
Non-Lassa fever patients^e^, n	304	304	304	200	200
Test positive, n	13	13	0	7	0
Test negative, n	291	291	304	193	200
Sensitivity, %	31.1	5.2	25.9	5.2	25.9
(95% CI)	(25.6–37.0)	(2.9–8.6)	(20.8–31.6)	(2.9–8.6)	(20.8–31.6)
Specificity, %	95.7	95.7	100	96.5	100
(95% CI)	(92.8–97.7)	(92.8–97.7)	(98.8–100)	(92.9–98.6)	(98.2–100)
Positive likelihood ratio	7.3	1.2	∞	1.5	∞
(95% CI)	(4.1–12.8)	(0.58–2.5)		(0.61–3.60)	
Negative likelihood ratio	0.72	0.99	0.74	0.98	0.74
(95% CI)	(0.66–0.78)	(0.95–1.03)	(0.69–0.79)	(0.95–1.02)	(0.69–0.79)

Abbreviations: 95% CI, 95% confidence interval.

^a^ Test positive: sample was IgM positive irrespective of IgG result.

^b^ Test positive: sample was IgM positive and IgG negative.

^c^ Test positive: sample was IgM positive and IgG positive.

^d^ RT-PCR-confirmed patients from Nigeria. The specimen that had been used to establish the diagnosis of Lassa fever by RT-PCR was tested in ELISA.

^e^ Patients from non-endemic areas Ghana (RT-PCR-negative) and Germany.

**Fig 4:**
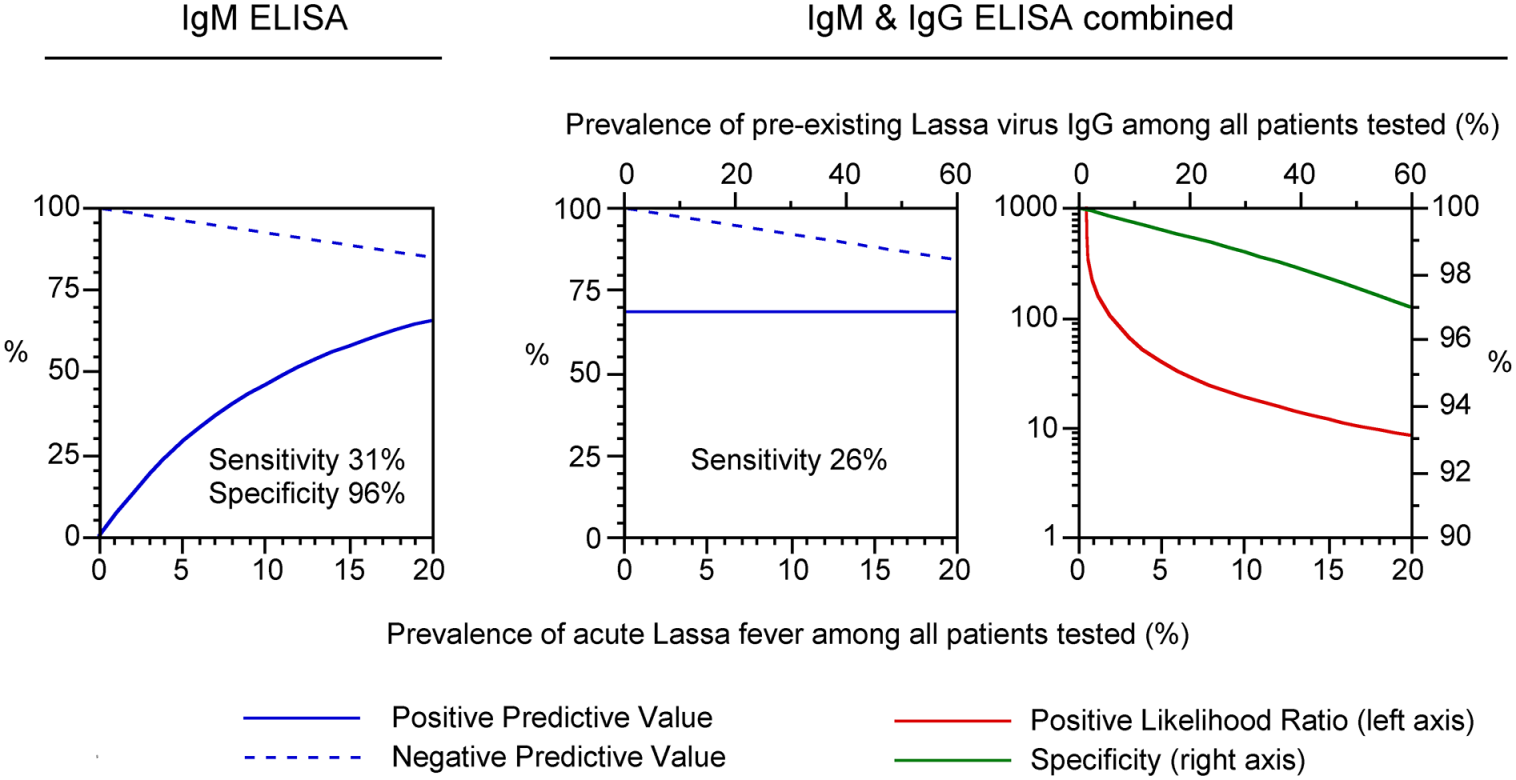
Clinical performance characteristics of the IgM ELISA as stand-alone test and in combination with the IgG ELISA depending on prevalence of Lassa fever and pre-existing IgG among all patients tested. The calculations are based on the data shown in Table 3. PPV and NPV of the stand-alone IgM ELISA depend only on the Lassa fever prevalence in diagnostics. However, PPV, NPV, sensitivity, and positive likelihood ratio for combined detection of IgM and IgG (i.e. a positive test result means that both IgM and IgG is positive) depend on the prevalence of Lassa fever as well as the prevalence of pre-existing IgG among all patients tested. To simplify calculation, we assumed a ratio of 1:3 between prevalence of Lassa fever and pre-existing IgG, which roughly corresponds to the setting in Nigeria where the study was performed.

PPV and NPV were calculated for a prevalence of Lassa fever patients among all patients tested ranging from 0% to 20% (Fig 4). The calculation for combined IgM and IgG detection also considered the impact of pre-existing IgG in the local population (IgG seroprevalence) on the diagnosis of acute Lassa fever. The PPV of a stand-alone IgM finding approximates zero at low prevalence and improves with increasing prevalence of Lassa fever cases, while combined detection of IgM and IgG shows a PPV of about 70% over the entire prevalence range. The NPV of the IgM assay alone and in combination with the IgG assay is 100% at low prevalence and decreases to 85% at high prevalence. With increasing prevalence of pre-existing IgG, specificity and positive likelihood ratio for combined IgM/IgG detection decrease from 100% to 97% and from ∞ to 10, respectively (Fig 4, right).

## Discussion

This study describes the establishment and evaluation of novel capture ELISA for detection of Lassa virus NP-specific IgM and IgG. The ELISAs detected Lassa virus-specific antibodies with high analytical accuracy. Evaluation of the ELISAs for diagnosis of acute Lassa fever revealed sensitivity for the stand-alone IgM ELISA of 31% and for combined IgM/IgG detection of 26%. Isolated IgM reactivity, i.e. IgM in the absence of IgG, has no diagnostic value. The clinical specificity of IgM and IgG ELISA was estimated at 96% and 100%, respectively.

In agreement with previous serological studies [20–22, 27–29], the ability of the ELISAs to diagnose Lassa fever at early stage is limited. The sensitivity of our stand-alone IgM ELISA is comparable to that of a previously published IgM ELISA, which detected 26% of virus culture-confirmed Lassa fever cases in the first specimen and 72% in all blood draws [22]. However, the sensitivity of combined IgM/IgG detection was lower than in our study.

Three main factors affected the performance of our assays. First, as most Lassa fever patients have not (yet) developed antibodies on admission to the hospital, the clinical sensitivity of IgM detection is low. Second, due to high IgG seroprevalence in endemic regions, the detection of IgG is not suitable for diagnosis of acute infection but improves performance in combination with the IgM assay, as discussed below. And third, we found evidence for false positive reactions in the IgM ELISA. Specificity issues are common to IgM detection assays and may stem from various conditions, including polyclonal B cell activation, vigorous immune response e.g. during acute malaria, or naturally occurring biotin IgM antibodies [41].

An observation that actually improves clinical performance of the ELISA was the rare occurrence of isolated IgM during early stage of disease. The majority of IgM positive patients already developed IgG. This is consistent with our follow-up data showing that Lassa virus-specific IgM and IgG develop simultaneously rather than sequentially. In some patients, IgG emerged even slightly earlier than IgM. The immunological mechanisms behind the rapid class switch from IgM to IgG or suppression of the IgM response are not known [42, 43]. On the other hand, this observation might be due to a higher analytical sensitivity of the IgG ELISA compared to the IgM ELISA. However, due to this specific feature, specificity, positive likelihood ratio, and PPV of the diagnostic method can be improved by combining IgM and IgG detection. The PPV of the stand-alone IgM assay, i.e. without considering IgG, strongly depends on the prevalence of Lassa fever among the hospital admissions. At low prevalence a stand-alone IgM finding is likely to be false positive. The additional information on the IgG status facilitates classification of IgM positive findings into two groups: (1) isolated IgM reactivity that is of no diagnostic value, and (2) IgM/IgG reactivity with a positive likelihood ratio >10 and a PPV around 70% independent of Lassa fever prevalence. This is in line with the improvement of the analytical performance (i.e. concurrence of the ELISA with IFA results) for detection of both IgM and IgG versus stand-alone or isolated IgM detection. In summary, due to the low sensitivity (26%) of IgM/IgG detection on admission, the ELISAs may play only a supplementary role in diagnostics of Lassa fever in the early stage. Even IgM/IgG-positive findings have to be interpreted with caution due to the moderate PPV (70%) of such finding. Importantly, our data confirm that the method of choice for early detection of Lassa fever is RT-PCR or sensitive antigen detection [12, 20–26].

A firm diagnosis of Lassa fever may be established by ELISA in patients in whom IgM and/or IgG seroconversion can be demonstrated. However, the window period before seroconversion delays diagnosis, and thus proper patient management, and fatal cases do not necessarily develop antibodies [27, 28]. The latter suggests that development of antibodies is a marker for a favorable prognosis, which might be of value in clinical management. The ELISAs may also have diagnostic value in Lassa fever patients presenting during convalescence after virus had been cleared, in patients with symptomatic virus persistence in CSF or other body fluids in the absence of viremia [44], or in patients with mild or asymptomatic infections [4], in whom the viremia might be below the detection limit of the PCR. Unfortunately, due to the lack of appropriate specimens, we cannot provide estimates for diagnostic accuracy of the ELISAs to detect these clinical conditions.

Another potential limitation of our study is that we have evaluated the assays in one endemic and two non-endemic areas only. The diagnostic accuracy of the assays might be somewhat different in other settings. Possible factors affecting performance include the average time between onset of Lassa fever and presentation at the hospital, the prevalence of Lassa fever, the ratio between clinical and subclinical Lassa virus infections, the prevalence of Lassa virus-specific antibodies in the population, and the frequency of cross-reactive antibodies or unspecific reactions. In order to generalize our data and provide estimates of performance in various settings, we have taken some of these factors into account for estimation of specificity, likelihood ratio, PPV and NPV. Nevertheless, before applying the ELISA in Lassa fever diagnostics it is essential that the performance characteristics be estimated—or even validated—according to the local conditions. Another factor influencing performance might be virus variability. The NP antigen used in the assays is derived from strain AV belonging to Lassa lineage IV circulating in Mali, Côte d’Ivoire, Guinea, Sierra Leone, and Liberia, while we evaluated the assay in an area where a heterologous lineage (II) is prevailing [6, 9, 10, 14, 38]. Therefore, we assume that the diagnostic accuracy in regions where other heterologous lineages circulate will essentially correspond to what we estimated here with lineage II-specific sera.

In addition to diagnostics in acute patients, the assays may be of value in epidemiological studies and clinical trials, e.g. to distinguish between natural immunity and immunity induced by glycoprotein-based vaccines [45]. The high specificity of the IgG ELISA, the clear distinction of negatives and positives in that assay via the S/CO ratio, and the higher throughput rate and easier operation compared to IFA will be of advantage in any kind of large-scale seroepidemiological study.

The established assays do not require expensive equipment; ELISA readers are available in many diagnostic laboratories in West Africa. The use of recombinant antigen will facilitate future assay production according to standards of good manufacturing practice and potentially commercialization. We are making efforts to provide the assays described here in industry-standard quality in future.

## Supporting information

S1 Checklist STARD checklist. The STARD 2015 checklist provides information on how this study complies with standards for reporting diagnostic accuracy studies.
(PDF)

S1 Diagram STARD flow diagram. The STARD 2015 flow diagram summarizes the criteria for selection of patients, definition of reference standard, and results of the index tests.
(PDF)

S1 Data Data from all experiments performed in this study. The spreadsheet contains a line list of all samples tested with the corresponding Lassa virus RT-PCR, IFA, and ELISA results. Note that some fields are interactive.
(XLSX)

S1 Fig Sample OD and cut-off values for individual ELISA plates. Each diagram shows the data obtained with one ELISA plate. The diagram title indicates the type of plate (RF IgG, CD32 IgG, or IgM), the origin of the samples (Nigeria, Ghana, or Germany), and the cut-off (CO) value. Each dot represents the optical density (OD) value of one serum sample. Samples from Nigeria are sorted according to the immunofluorescence (IFA) categories "clearly negative" (0; circles), "probable positive" (0.5, squares), and "clearly positive" (1, triangles). Cut-off values for all plates were calculated with the formula *Cut-off = 3 × Mean OD of negative standards + 0*.*06*. The mean of the negative standards and the cut-off value are indicated by horizontal dotted lines in black and red, respectively.
(PDF)
