# Supplementary material for: Development and evaluation of antibody-capture immunoassays for detection of Lassa virus nucleoprotein-specific immunoglobulin M and G
Source: PLoS Negl Trop Dis. 2018 Mar 29;12(3):e0006361. doi: 10.1371/journal.pntd.0006361 (PMC5892945; doi:10.1371/journal.pntd.0006361)
Supplement: S1 Checklist — The STARD 2015 checklist provides information on how this study complies with standards for reporting diagnostic accuracy studies. (PDF) [file pntd.0006361.s001.pdf]

Section & Topic	No	Item	Reported on page #
**TITLE OR ABSTRACT**			
	**1**	Identification as a study of diagnostic accuracy using at least one measure of accuracy (such as sensitivity, specificity, predictive values, or AUC)	Accuracy, sensitivity, and specificity data are mentioned in the Abstract.
**ABSTRACT**			
	**2**	Structured summary of study design, methods, results, and conclusions (for specific guidance, see STARD for Abstracts)	Abstract is structured and provides conclusions regarding performance and potential applications of the index test.
**INTRODUCTION**			
	**3**	Scientific and clinical background, including the intended use and clinical role of the index test	Background on Lassa fever and the intended use of the index test in diagnostics of Lassa fever is provided in the Introduction.
	**4**	Study objectives and hypotheses	The aim of the study is stated at the end of the Introduction
**METHODS**			
Study design	**5**	Whether data collection was planned before the index test and reference standard were performed (prospective study) or after (retrospective study)	The study was performed retrospectively with respect to the collection of specimens (see "Patients and specimens").
Participants	**6**	Eligibility criteria	The specimens/participants were randomly chosen from the available specimen pools (see "Patients and specimens").
	**7**	On what basis potentially eligible participants were identified (such as symptoms, results from previous tests, inclusion in registry)	Specimens/participants were selected based on previous results in RT-PCR (positive or negative) and based on country of origin (endemic or non-endemic areas). To provide a more meaningful sample for the purpose of this study, patients who tested positive in RT-PCR were intentionally overrepresented (see "Patients and specimens").
	**8**	Where and when potentially eligible participants were identified (setting, location and dates)	Settings, locations, and dates of sampling are provided (see "Patients and specimens").
	**9**	Whether participants formed a consecutive, random or convenience series	Specimens/participants from each setting form a consecutive series (see "Patients and specimens").
Test methods	**10a**	Index test, in sufficient detail to allow replication	The in-house preparation and implementation of the index tests (ELISA) is described in detail (see "Expression and purification of recombinant NP" and "Lassa virus NP-specific IgG and IgM ELISA").
	**10b**	Reference standard, in sufficient detail to allow replication	The clinical reference standard was RT-PCR (for presence of target condition) in combination with origin of patient (for absence of target condition) (see "Patients and specimens"). The RT-PCR has been described previously and literature is cited. The analytical reference standard (IFA) is described in "Lassa virus-specific IFA".
	**11**	Rationale for choosing the reference standard (if alternatives exist)	The clinical reference standard for presence of disease (RT-PCR) is the method of choice for early diagnosis of Lassa fever. Origin of patient from a non-endemic country in combination with negative RT-PCR was chosen as reference to define absence of disease, as this is more accurate in ruling out Lassa virus infection than a negative RT-PCR result in a patient from endemic area. The analytical reference standard (IFA) is the classical and most widely used test for detection of Lassa virus-specific antibodies (section "Patients and specimens").
	**12a**	Definition of and rationale for test positivity cut-offs or result categories of the index test, distinguishing pre-specified from exploratory	The cut-off for the index test was calculated using the equation: $\textit{Cut-off} = a \times \textit{Mean OD of negative standards} + b$. After all data had been collected, we found empirically that $a = 3$ and $b = 0.06$ facilitate good correlation of the ELISA with the IFA results, in particular for sera classified as "clearly negative" and "clearly positive" in IFA (see "Lassa virus NP-specific IgG and IgM ELISA").
	**12b**	Definition of and rationale for test positivity cut-offs or result categories of the reference standard, distinguishing pre-specified from exploratory	The interpretation of Lassa virus RT-PCR results had been done according to common laboratory practice. The details are published. Endemic and non-endemic areas for Lassa fever were defined according to currently available epidemiological information. IFA signals were evaluated by fluorescence microscopy and classified as "clearly negative", "probable positive", and "clearly positive" by the investigator (see "Lassa virus-specific IFA").

CC BY SA

	**13a**	Whether clinical information and reference standard results were available to the performers/readers of the index test	The investigator knew the origin of the samples during assay evaluation, though not the RT-PCR result. IFA and ELISA were performed in a blinded fashion, i.e. the investigator did not know the corresponding assay result. Samples from each setting were tested consecutively according to the identification number. Diagnosis, IFA result, and ELISA result were linked after testing (section "Patients and specimens").
	**13b**	Whether clinical information and index test results were available to the assessors of the reference standard	As the study was performed retrospectively, the index test results were not known to assessors of the reference standard (see "Patients and specimens").
Analysis	**14**	Methods for estimating or comparing measures of diagnostic accuracy	Methods for estimating analytical and clinical diagnostic accuracy are described in "Calculation of performance characteristics".
	**15**	How indeterminate index test or reference standard results were handled	The index test had no indeterminate results. For calculation of analytical performance characteristics of the ELISA compared to IFA, the "probable positive" and "clearly positive" IFA categories were merged into one IFA "positive" category (see Results). Patients with inconclusive Lassa fever status were excluded.
	**16**	How missing data on the index test and reference standard were handled	There were no missing data on the index test and reference standard.
	**17**	Any analyses of variability in diagnostic accuracy, distinguishing pre-specified from exploratory	Variability in diagnostic accuracy was estimated depending on pre-specified prevalence of Lassa fever and Lassa virus-specific IgG antibodies (see Figure 4).
	**18**	Intended sample size and how it was determined	We aimed at a sample size of approximately 300 patients per group, as this number facilitates detection of small proportions at reasonable precision, for example a 2%-false positive rate with specified limits of the 95% confidence interval at 0.5% and 3.5% (see "Patients and specimens").
**RESULTS**			
Participants	**19**	Flow of participants, using a diagram	See S1 Diagram.
	**20**	Baseline demographic and clinical characteristics of participants	Information on patients was limited, as the study relied to large extent on anonymized diagnostic leftover specimens. The origin of patients on country level is provided (see "Patients and specimens").
	**21a**	Distribution of severity of disease in those with the target condition	The study distinguished only between Lassa fever and non-Lassa fever. Clinical details on severity of Lassa fever were not known.
	**21b**	Distribution of alternative diagnoses in those without the target condition	Non-Lassa patients from Ghana were suspected to have viral hemorrhagic fever or viral hepatitis. The diagnoses of non-Lassa patients from Germany were not known (see "Patients and specimens").
	**22**	Time interval and any clinical interventions between index test and reference standard	The index test and reference standards were performed retrospectively on the same specimens. Thus, there was no time interval (see "Patients and specimens").
Test results	**23**	Cross tabulation of the index test results (or their distribution) by the results of the reference standard	Cross tabulation of the test results by the results of analytical and clinical reference standards are provided in Tables 1 and 3, respectively.
	**24**	Estimates of diagnostic accuracy and their precision (such as 95% confidence intervals)	Estimates of diagnostic accuracy are provided in Table 1 and 3. 95% confidence intervals are given in Table 3.
	**25**	Any adverse events from performing the index test or the reference standard	Index test and reference standards were performed on blood of patients. Adverse events due to blood sampling were not reported.
**DISCUSSION**			
	**26**	Study limitations, including sources of potential bias, statistical uncertainty, and generalisability	We have evaluated our assays in one endemic and two non-endemic areas. It is conceivable that the diagnostic accuracy of the assays is different in other settings. In order to generalize our data, we have taken into account local conditions in estimation of specificity, likelihood ratio, PPV and NPV (see Figure 4 and Discussion).
	**27**	Implications for practice, including the intended use and clinical role of the index test	These aspects are extensively discussed at the end of the Discussion.
**OTHER INFORMATION**			
	**28**	Registration number and name of registry	None
	**29**	Where the full study protocol can be accessed	Not applicable, as the study relied to large extent on anonymized diagnostic leftover specimens.
	**30**	Sources of funding and other support; role of funders	Is provided in the funding statement.

STARD 2015

AIM

STARD stands for "Standards for Reporting Diagnostic accuracy studies". This list of items was developed to contribute to the completeness and transparency of reporting of diagnostic accuracy studies. Authors can use the list to write informative study reports. Editors and peer-reviewers can use it to evaluate whether the information has been included in manuscripts submitted for publication.

EXPLANATION

A **diagnostic accuracy study** evaluates the ability of one or more medical tests to correctly classify study participants as having a **target condition.** This can be a disease, a disease stage, response or benefit from therapy, or an event or condition in the future. A medical test can be an imaging procedure, a laboratory test, elements from history and physical examination, a combination of these, or any other method for collecting information about the current health status of a patient.

The test whose accuracy is evaluated is called **index test.** A study can evaluate the accuracy of one or more index tests. Evaluating the ability of a medical test to correctly classify patients is typically done by comparing the distribution of the index test results with those of the **reference standard**. The reference standard is the best available method for establishing the presence or absence of the target condition. An accuracy study can rely on one or more reference standards.

If test results are categorized as either positive or negative, the cross tabulation of the index test results against those of the reference standard can be used to estimate the **sensitivity** of the index test (the proportion of participants *with* the target condition who have a positive index test), and its **specificity** (the proportion *without* the target condition who have a negative index test). From this cross tabulation (sometimes referred to as the contingency or "2x2" table), several other accuracy statistics can be estimated, such as the positive and negative **predictive values** of the test. Confidence intervals around estimates of accuracy can then be calculated to quantify the statistical **precision** of the measurements.

If the index test results can take more than two values, categorization of test results as positive or negative requires a **test positivity cut-off**. When multiple such cut-offs can be defined, authors can report a receiver operating characteristic (ROC) curve which graphically represents the combination of sensitivity and specificity for each possible test positivity cut-off. The **area under the ROC curve** informs in a single numerical value about the overall diagnostic accuracy of the index test.

The **intended use** of a medical test can be diagnosis, screening, staging, monitoring, surveillance, prediction or prognosis. The **clinical role** of a test explains its position relative to existing tests in the clinical pathway. A replacement test, for example, replaces an existing test. A triage test is used before an existing test; an add-on test is used after an existing test.

Besides diagnostic accuracy, several other outcomes and statistics may be relevant in the evaluation of medical tests. Medical tests can also be used to classify patients for purposes other than diagnosis, such as staging or prognosis. The STARD list was not explicitly developed for these other outcomes, statistics, and study types, although most STARD items would still apply.

DEVELOPMENT

This STARD list was released in 2015. The 30 items were identified by an international expert group of methodologists, researchers, and editors. The guiding principle in the development of STARD was to select items that, when reported, would help readers to judge the potential for bias in the study, to appraise the applicability of the study findings and the validity of conclusions and recommendations. The list represents an update of the first version, which was published in 2003.

More information can be found on http://www.equator-network.org/reporting-guidelines/stard.

CC BY SA
