# Supplementary figures and images for: Development and evaluation of antibody-capture immunoassays for detection of Lassa virus nucleoprotein-specific immunoglobulin M and G
Source: PLoS Negl Trop Dis. 2018 Mar 29;12(3):e0006361. doi: 10.1371/journal.pntd.0006361 (PMC5892945; doi:10.1371/journal.pntd.0006361)

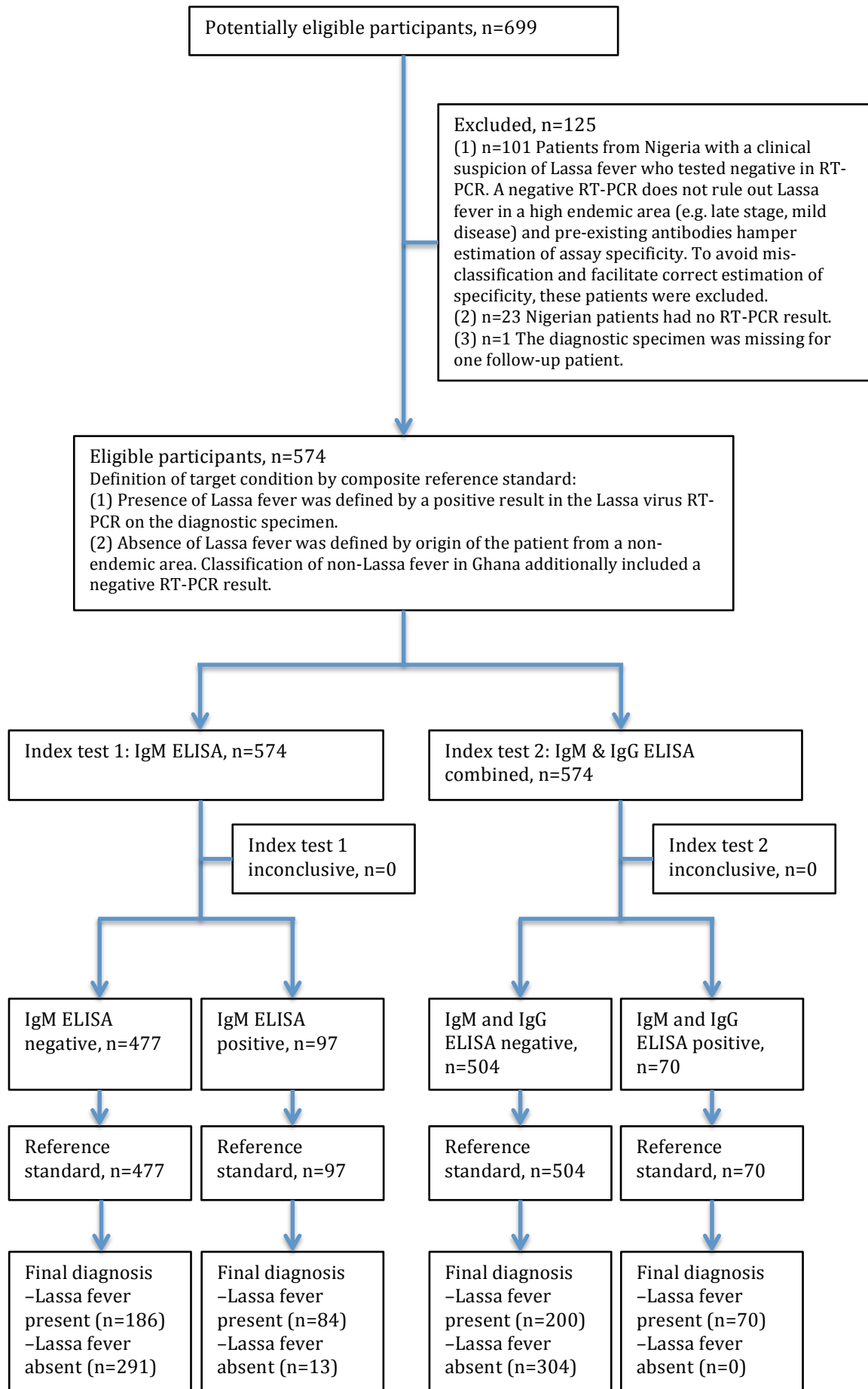

Supplement: S1 Diagram — The STARD 2015 flow diagram summarizes the criteria for selection of patients, definition of reference standard, and results of the index tests. (PDF) [file pntd.0006361.s002.pdf]

# Cut-off for IgM ELISA plates

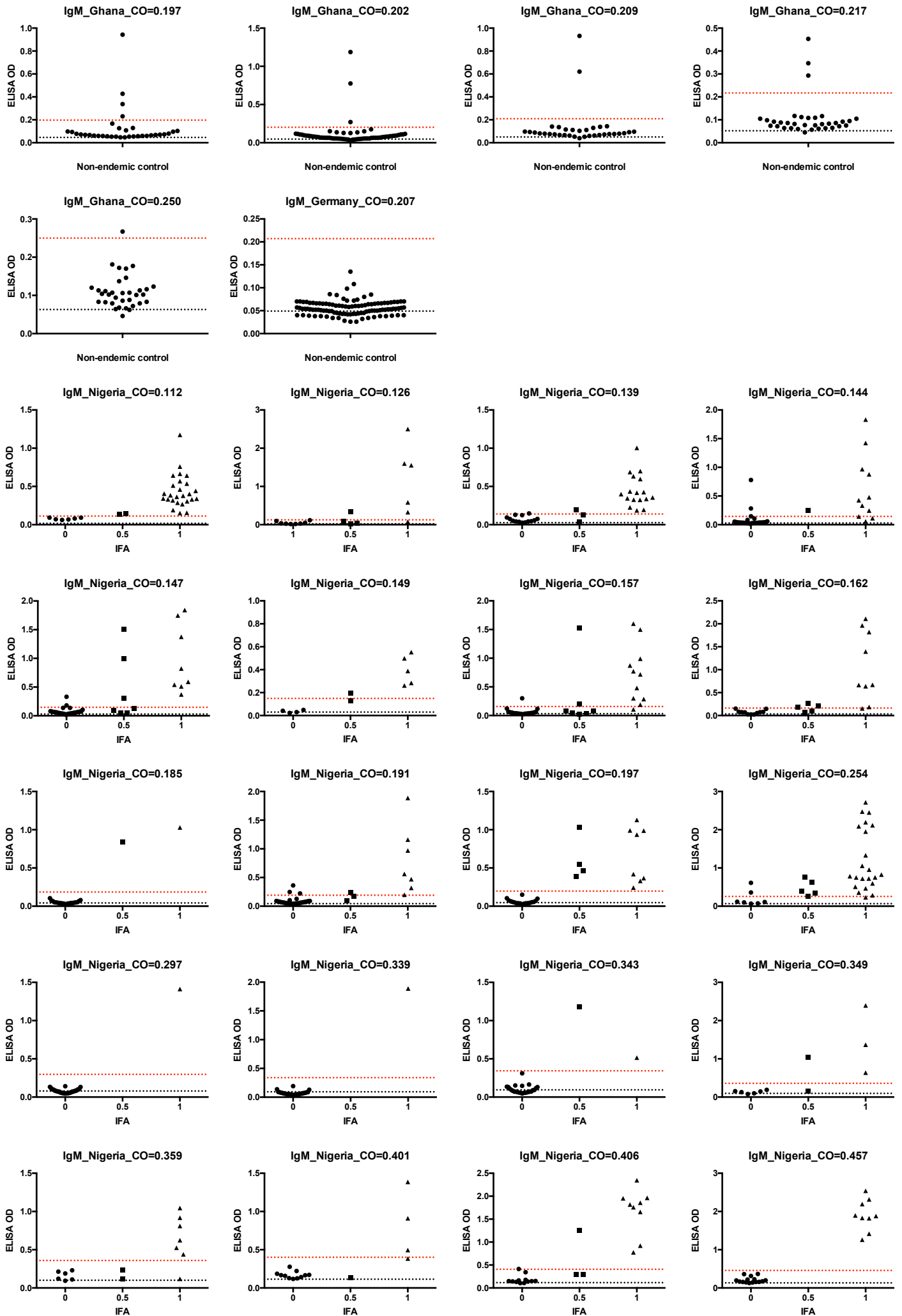

# Cut-off for RF IgG ELISA plates

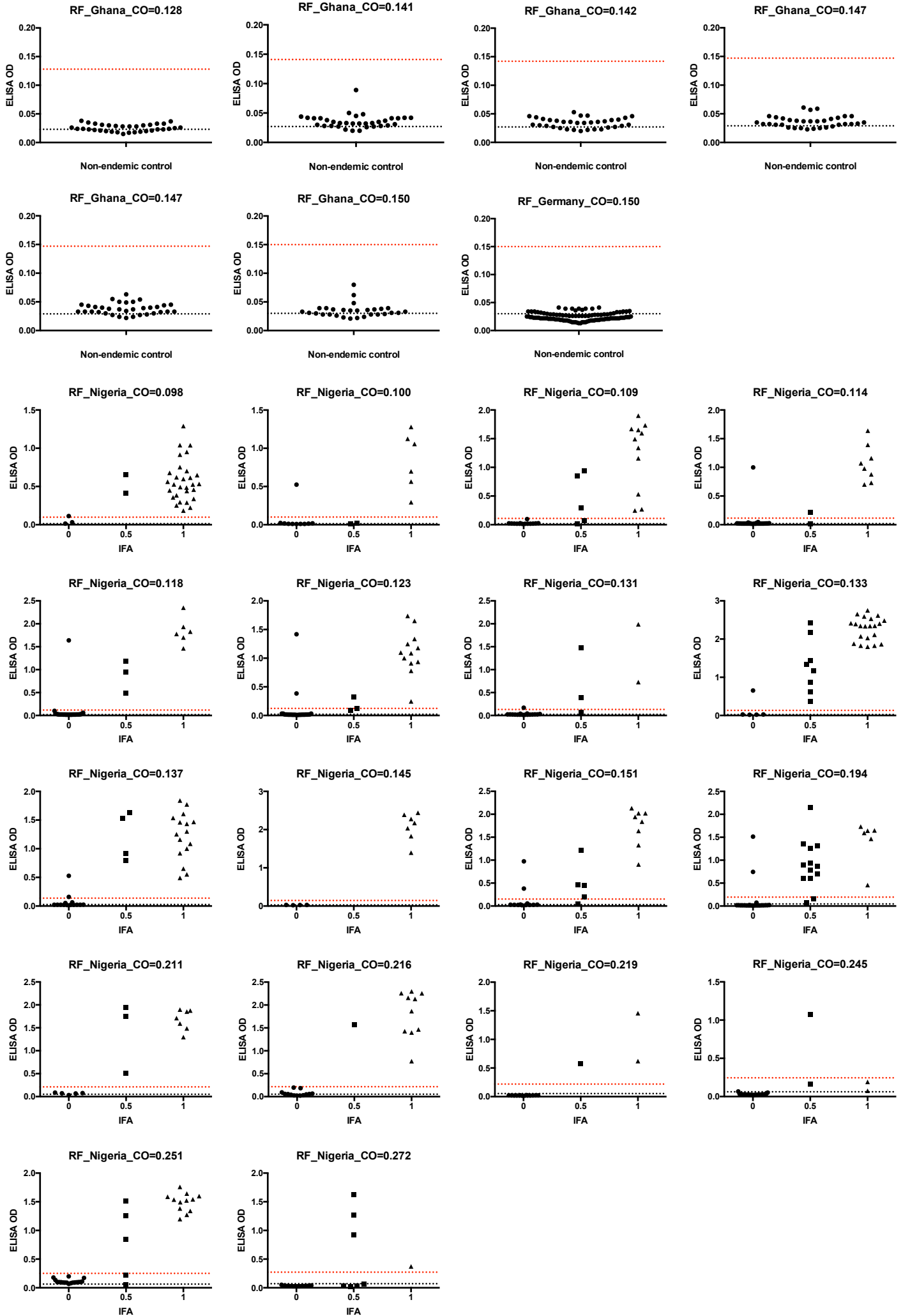

# Cut-off for CD32 IgG ELISA plates

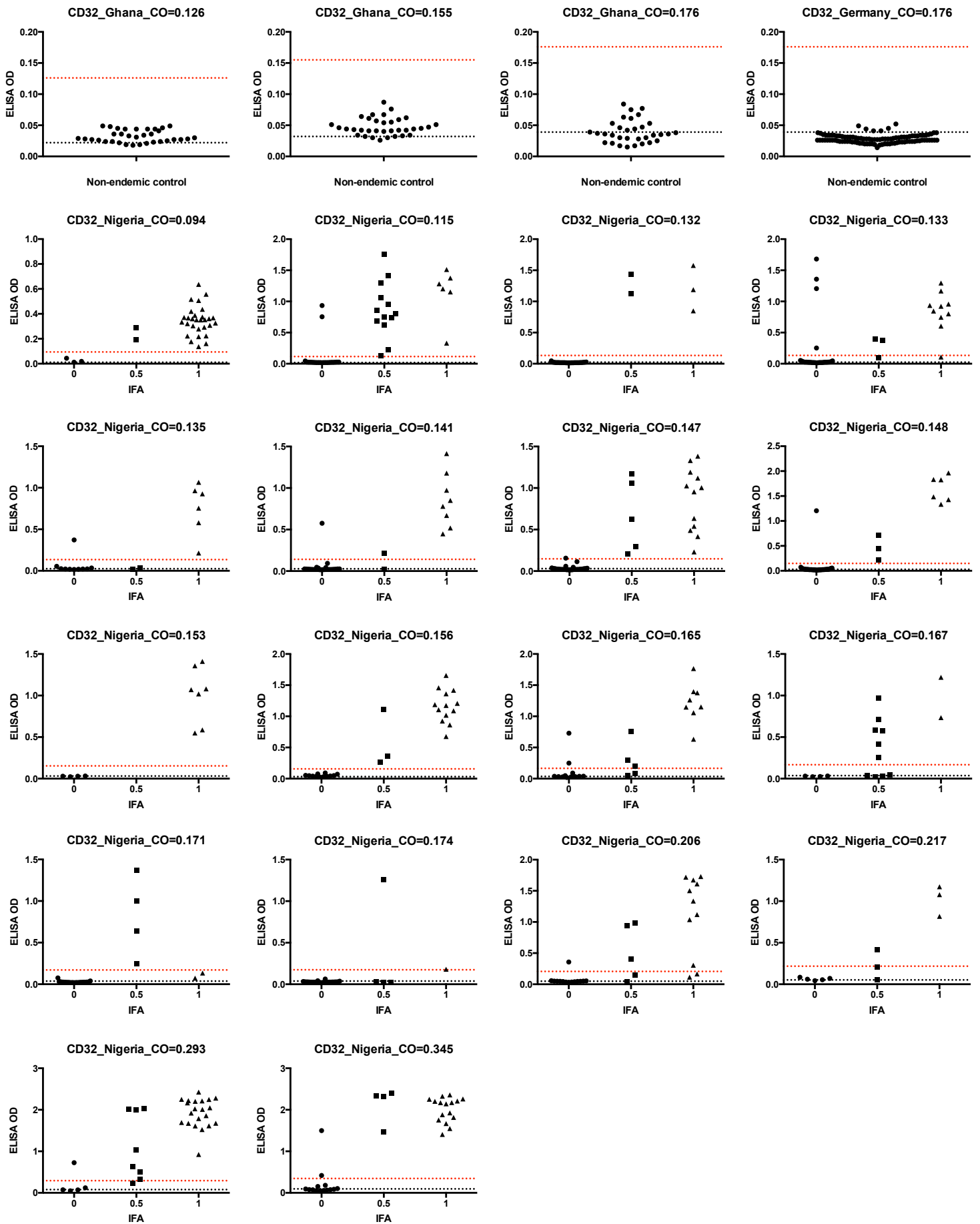

Supplement: S1 Fig — Each diagram shows the data obtained with one ELISA plate. The diagram title indicates the type of plate (RF IgG, CD32 IgG, or IgM), the origin of the samples (Nigeria, Ghana, or Germany), and the cut-off (CO) value. Each dot represents the optical density (OD) value of one serum sample. Samples from Nigeria are sorted according to the immunofluorescence (IFA) categories "clearly negative" (0; circles), "probable positive" (0.5, squares), and "clearly positive" (1, triangles). Cut-off values for all plates were calculated with the formula Cut-off = 3 × Mean OD of negative standards + 0.06. The mean of the negative standards and the cut-off value are indicated by horizontal dotted lines in black and red, respectively. (PDF) [file pntd.0006361.s004.pdf]
